# Early response to neoadjuvant chemotherapy can help predict long-term survival in patients with cervical cancer

**DOI:** 10.18632/oncotarget.11460

**Published:** 2016-08-20

**Authors:** Xiong Li, Kecheng Huang, Qinghua Zhang, Jian Shen, Hang Zhou, Runfeng Yang, Lin Wang, Jiong Liu, Jincheng Zhang, Haiying Sun, Yao Jia, Xiaofang Du, Haoran Wang, Song Deng, Ting Ding, Jingjing Jiang, Yunping Lu, Shuang Li, Shixuan Wang, Ding Ma

**Affiliations:** ^1^ Department of Obstetrics and Gynecology, Tongji Hospital, Tongji Medical College, Huazhong University of Science and Technology, Wuhan, Hubei, China; ^2^ Department of Obstetrics and Gynecology, Wuhan Central Hospital, Wuhan, Hubei, China; ^3^ Department of Obstetrics and Gynecology, Nanjing Drum Tower Hospital, The Affiliated Hospital of Nanjing University Medical School, Nanjing, Jiangsu, China; ^4^ Hubei Tumor Hospital, Wuhan, Hubei, China; ^5^ Shanghai Jiao Tong University School of Medicine, Shanghai, China; ^6^ Xinhua Hospital, Shanghai Jiao Tong University School of Medicine, Shanghai, China; ^7^ Department of Internal Medicine, Luohe Renmin Hospital, The First Affiliated Hospital of Luohe Medical College, Luohe, Henan, China; ^8^ Department of Obstetrics and Gynecology, University Hospital of Hubei University for Nationalities, Enshi, Hubei, China

**Keywords:** cervical cancer, clinical response, neoadjuvant chemotherapy (NACT), predictor, disease-free survival (DFS)

## Abstract

It is still controversial whether cervical cancer patients with clinical responses after neoadjuvant chemotherapy (NACT) have a better long-term survival or not. This study was designed to investigate the effect of the clinical response on the disease-free survival (DFS) of cervical cancer patients undergoing NACT. A total of 853 patients from a retrospective study were used to evaluate whether the clinical response was an indicator for the long-term response, and 493 patients from a prospective cohort study were used for further evaluation. The survival difference was detected by log-rank test, univariate and multivariate Cox regression and a pooled analysis. The log-rank test revealed that compared with non-responders, the DFS of responders was significantly higher in the retrospective data (P = 0.007). Univariate Cox regression showed that the clinical response was an indicator of long-term survival in the retrospective study (HR 1.83, 95% CI 1.18-2.85, P = 0.007). In a multivariate Cox model, the clinical response was still retained as an independent significant prognostic factor in the retrospective study (HR 1.59, 95% CI 1.01-2.50, P = 0.046). The result was also validated in the prospective data with similar results. These findings implied that the clinical response can be regarded as an independent predictor of DFS.

## INTRODUCTION

Cervical cancer is the second common malignant tumor-causing disease in women in undeveloped countries [1]. East Asia contributes more than 12% of new global cases or deaths [2, 3], and more than 130,000 new patients and 50,000 deaths each year [4]. As demonstrated by the National Comprehensive Cancer Network (NCCN) guidelines and the International Federation of Gynecology and Obstetrics (FIGO) system, neoadjuvant chemotherapy (NACT) plus surgery has emerged as a promising therapy for locally advanced cervical cancer. First, NACT can help reduce tumor size, diminish the expansion of tumors [5], and eliminate distant metastasis [6–9]. Consequently, more patients get the chance for surgery, leading to great improvements in their quality of life [10]; both young and old women benefit from NACT plus surgery, as young women can have their fertility preserved [11, 12], pre-menopausal women can have their sexual function preserved, and old women can get avoid the severe side effects of radiation therapy. Meanwhile, previous studies have also demonstrated that long-term survival may be significantly increased by NACT plus surgery compared with surgery alone or radiation therapy [6, 13, 14].

However, it is still controversial whether cervical cancer patients with clinical responses after NACT have better long-term survival. Many studies have shown that the clinical response is not a significant prognostic predictor or that it may not have an effect on survival [15–18], although our previous study revealed that clinical responders had a higher survival rate than non-responders [19].

The sample size of the previous studies was relatively small; thus, bias may exist. This study was designed to test whether the clinical response to NACT was a predictor of long-term survival among patients with cervical cancer in a large retrospective study; meanwhile, data from a prospective cohort were also used for further validation.

## RESULTS

In the retrospective analysis, we included 853 patients with stage IB2-IIB cervical cancer receiving neo-adjuvant platinum-based chemotherapy and radical hysterectomy (Table 1). The median age of the patients at the time of study entry was 44 (range 39-50) years. In the prospective cohort, which was also used in the previous study, 493 patients were included in the beginning, all of whom underwent neo-adjuvant platinum-based chemotherapy and radical hysterectomy (Supplementary Figure 1); the details are listed in Table 1.

**Table 1:** Clinical characteristics of all patients

Characteristics	Retrospective (n = 853)		Prospective (n = 493)	
	No.	%	No.	%
Age (25th-75th percentiles) (year)				
Median	44		45	
Range	39-50		40-49	
Tumor size (25th-75th percentiles) (cm)				
Median	4.0 3.5-5.0		4.0	
Range			3.0-5.0	
Tumor grade				
G1	58	6.8	36	7.3
G2	354	41.5	221	44.8
G3	240	28.1	162	32.9
Undetermined	201	23.6	74	15.0
FIGO stage				
IB2	220	25.8	127	25.8
IIA	265	31.1	117	23.7
IIB	368	43.1	249	50.5
Cell type				
Squamous	756	88.6	436	88.4
Non-squamous Unknown	91 6	10.7 0.7	53 4	10.8 0.8

FIGO, International Federation of Gynecology and Obstetrics.

### Log-rank test

The log-rank test was used to compare the difference in survival between the responders and non-responders, while the Kaplan-Meier method was used to draw a survival curve. The results revealed that the responders had significantly superior survival to the non-responders (P = 0.007 for DFS in the retrospective study and P = 0.004 for DFS in the prospective study), and the Kaplan-Meier plot also showed that the responders had higher survival rates than the non-responders.

### Univariate Cox regression analysis

In both the retrospective study and the prospective cohort study, the difference in survival was compared between the responders and non-responders. A Cox proportional hazard regression model was used to evaluate the effect of the risk factors effect on survival; the potential risk factors included clinical response, age, tumor size, FIGO stage, cell type, grade, lymph vascular space invasion (LVSI), parametrial infiltration, vaginal surgical margin and lymph node metastasis. A forest plot was employed to illustrate the HR and its 95% CI based on univariate Cox regression analysis. In the retrospective study, the clinical response was an indicator of DFS with statistical significance as the responders had superior survival rates with a HR of 1.83 (95% CI 1.18 to 2.85; P = 0.007) compared with the non-responders (Figure 2). In the prospective cohort study (Figure 3), the clinical response was also a significant indicator of DFS (HR 2.50; 95% CI 1.44 to 4.34; P = 0.001). More details are listed in Supplementary Table 2 and Supplementary Table 3.

**Figure 1:**
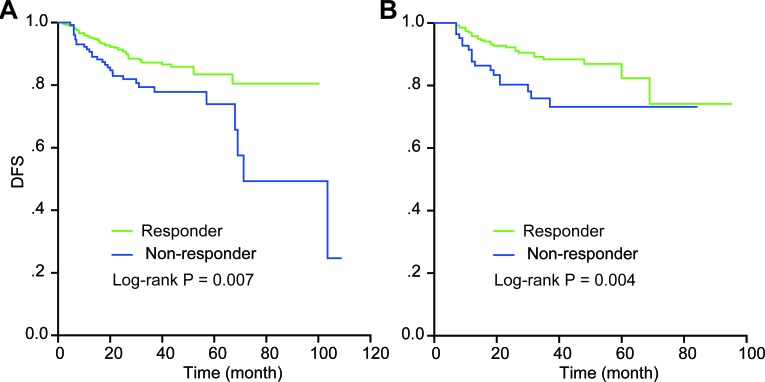
Kaplan-Meier survival estimates for responders and non-responders in the retrospective study and the prospective study Disease-free survival (DFS) curves of the evaluated patients in the retrospective study **A.** and the prospective cohort **B.** are shown. A log-rank test used to calculate the *P* values. Statistical significances were observed between the responders and the non-responders.

**Figure 2:**
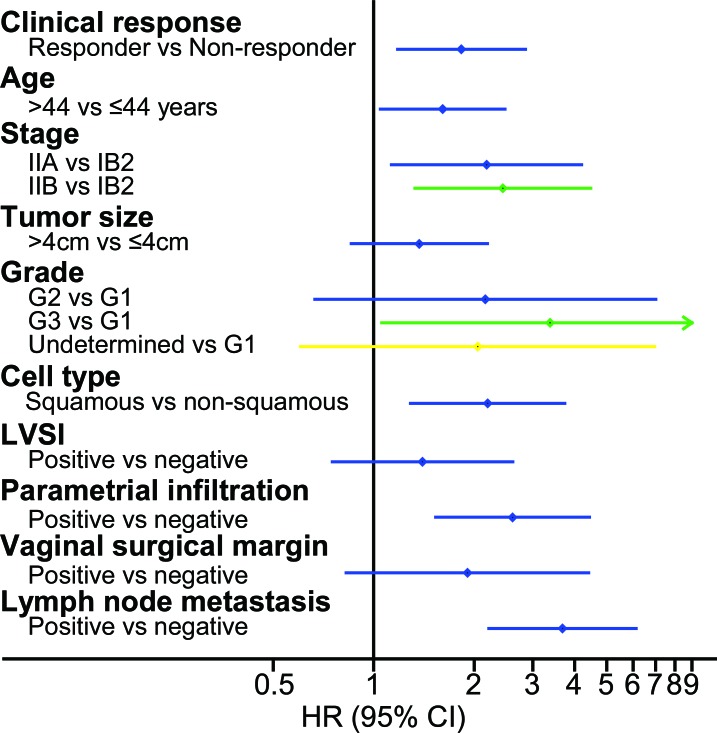
Univariate prognostic factor analysis in the retrospective study Univariate Cox analysis showed that several factors were significantly associated with DFS, including clinical response, age, FIGO stage, grade (G3 vs G1), cell type, parametrial infiltration and lymph node metastasis. Abbreviations: HR, hazard ratio; FIGO, International Federation of Gynecology and Obstetrics; DFS, disease-free survival; LVSI, lymph vascular space invasion.

**Figure 3:**
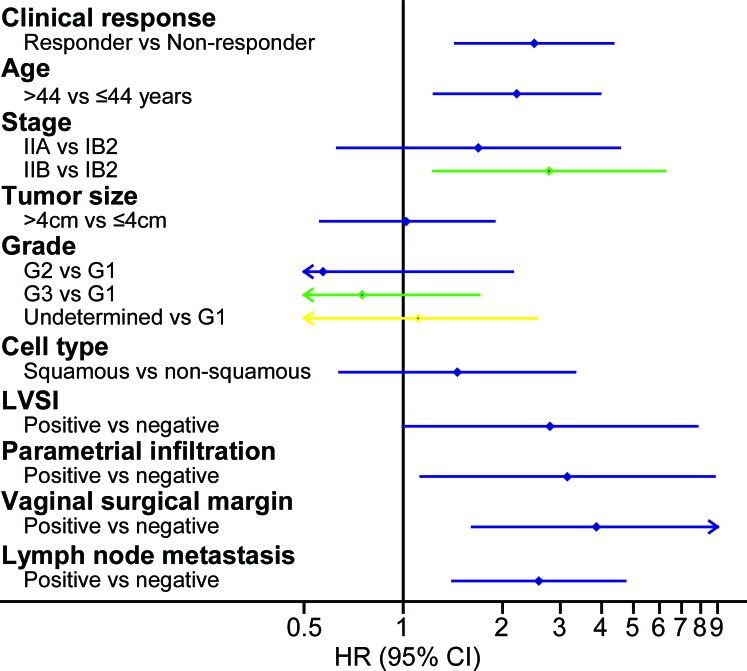
Multivariate prognostic factor analysis in the retrospective study Multivariate Cox analysis showed that several factors were significantly associated with DFS, including clinical response, FIGO stage, grade (G3 vs G1), cell type, parametrial infiltration and lymph node metastasis. Abbreviations: HR, hazard ratio; FIGO, International Federation of Gynecology and Obstetrics; DFS, disease-free survival; LVSI, lymph vascular space invasion.

### Multivariate Cox regression analysis

**Figure 4:**
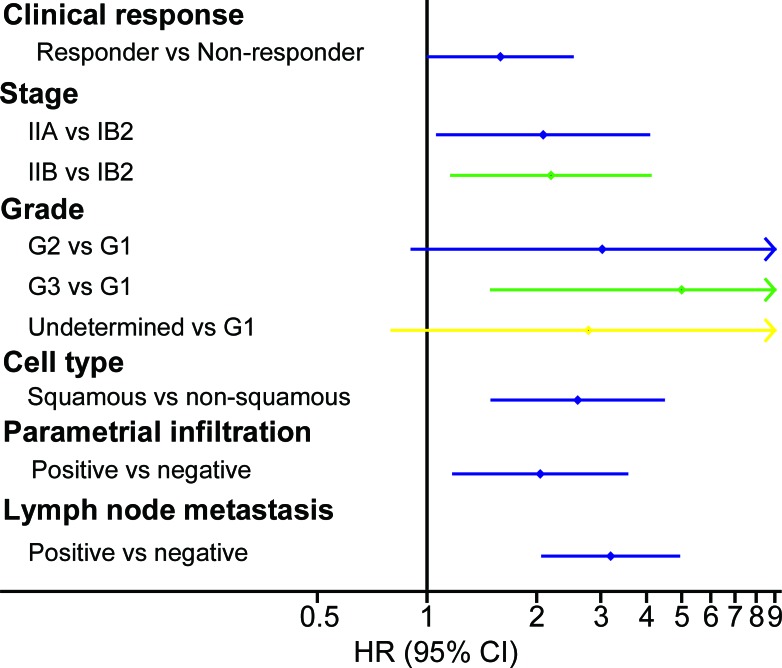
Univariate prognostic factor analysis in the prospective study Univariate Cox analysis showed that several factors were significantly associated with DFS, including clinical response, age, FIGO stage, parametrial infiltration, vaginal surgical margin and lymph node metastasis. Abbreviations: HR, hazard ratio; FIGO, International Federation of Gynecology and Obstetrics; DFS, disease-free survival; LVSI, lymph vascular space invasion.

**Figure 5:**
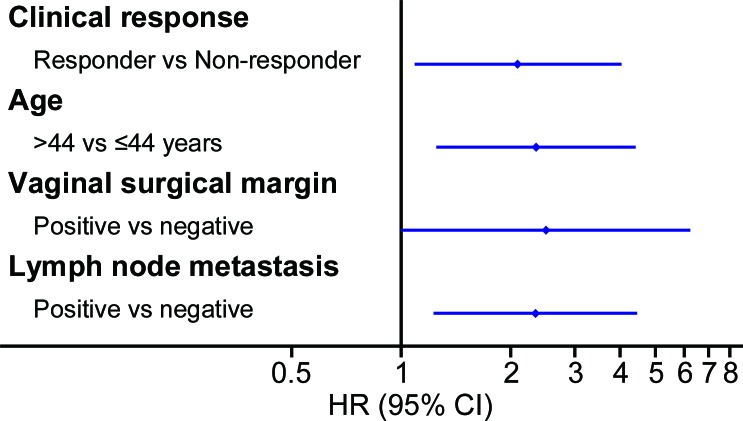
Multivariate prognostic factor analysis in the prospective study Multivariate Cox analysis showed that several factors were significantly associated with DFS, including clinical response, age, vaginal surgical margin and lymph node metastasis. Abbreviations: HR, hazard ratio; FIGO, International Federation of Gynecology and Obstetrics; DFS, disease-free survival; LVSI, lymph vascular space invasion.

Multivariate Cox proportional hazard regression was used to evaluate whether the clinical response was an independent prognostic factor for long-term survival. With similar methods as those used above, all the risk factors, including clinical-response, were assessed in the retrospective study (Figure 4) and in the prospective cohort (Figure 5). The results showed that compared with the non-responders, the responders had superior survival rates after adjustment with all the risk factors; the factor had a HR of 1.59 for DFS (95% CI 1.01 to 2.50; P = 0.046) in the retrospective study (Supplementary Table 4) and a HR of 2.09 for DFS (95% CI 1.10 to 4.00; P = 0.02) in the prospective cohort (Supplementary Table 5).

### Combined results of Cox analysis of the clinical response

**Figure 6:**
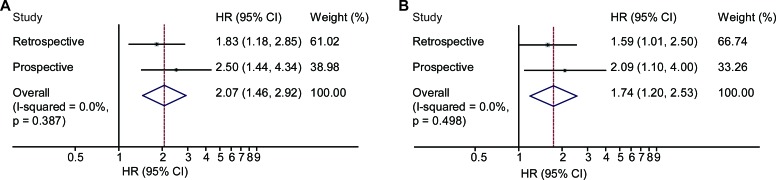
The combined clinical response results in both of the studies Combined Cox analysis showed that the clinical response was significantly associated with DFS. **A.** meta-analysis for univariate Cox analysis; **B.** meta-analysis for multivariate Cox analysis.

The results from the retrospective study and the prospective study were combined together according to the method illustrated in the previous study [20]. In univariate Cox analysis, the HR was 2.07 (95% CI, 1.46 to 2.92) (Figure 6A). In multivariate Cox analysis, the HR was 1.74 (95% CI, 1.20 to 2.53) (Figure 6B).

### Survival difference in each FIGO stage

**Figure 7:**
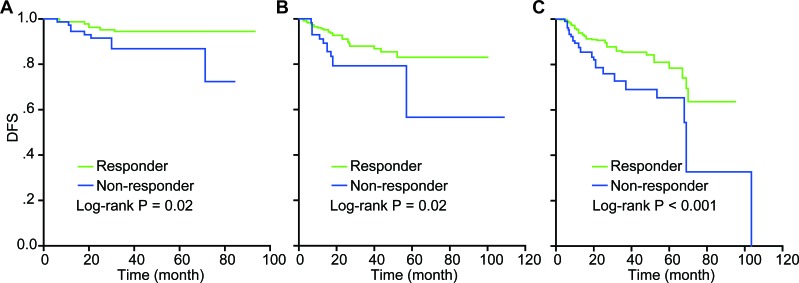
The DFS difference in each FIGO stage according to clinical response A log-rank test showed that DFS in the responder group was significantly higher than in the non-responder group: **A.** *P* = 0.02 for FIGO IB2; **B.** *P* = 0.02 for FIGO IIA; C. *P* < 0.001 for FIGO IIB. *P* < 0.05 indicates statistical significance.

After the data from the two studies was combined, a log-rank test was used to compare the difference in DFS between the responder and the non-responder groups for each FIGO stage; meanwhile, the Kaplan-Meier method was used to draw a survival curve. The results revealed that responders had superior survival to non-responders with statistical significance in each stage (P < 0.05) as the Kaplan-Meier plot also showed that the responders had higher disease-free survival rates than non-responders (Figure 7).

### High-risk prognostic factors’ distribution among responders and non-responders

**Figure 8:**
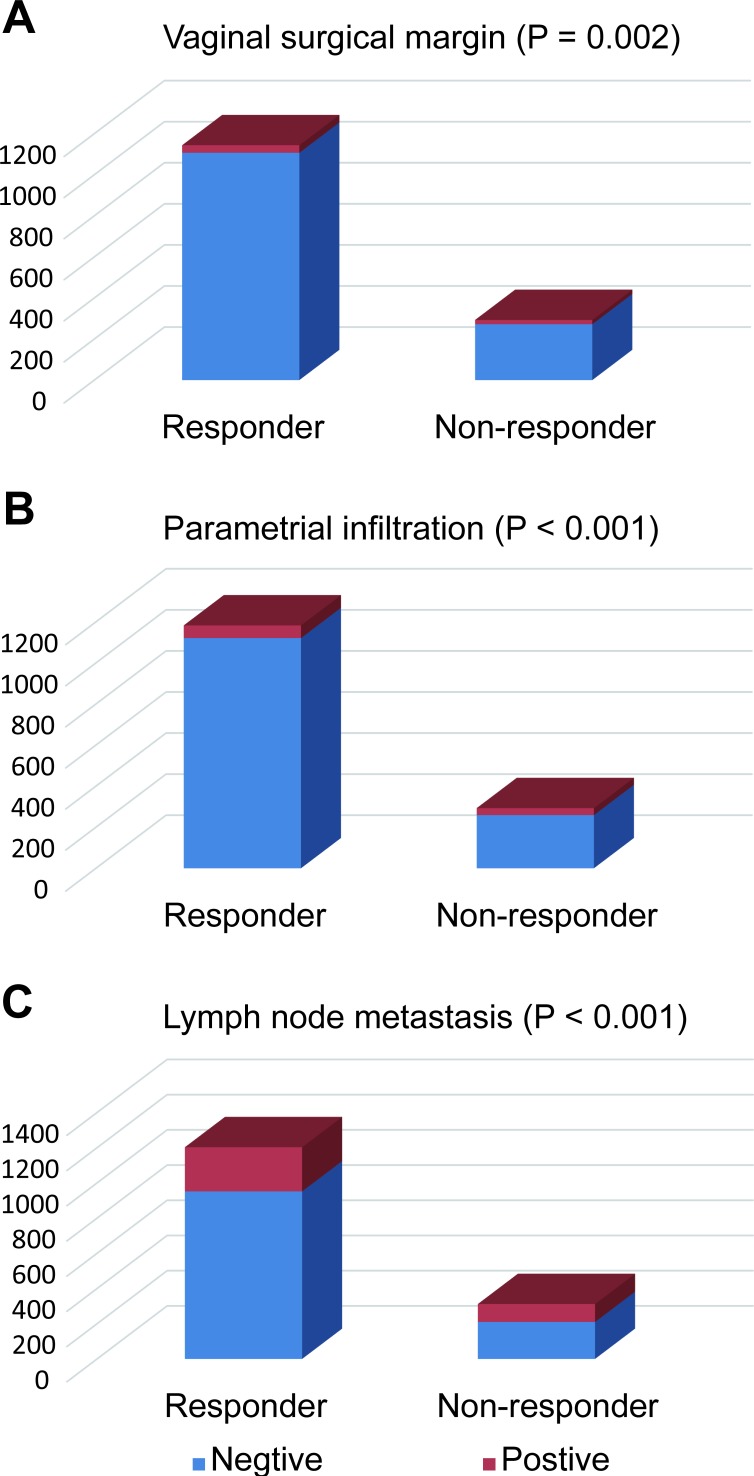
High-risk prognostic factors’ distribution among the responders and non-responders The ratio of cases with **A.** positive vaginal surgical margin (*P* = 0.002), **B.** positive parametrial infiltration (*P* < 0.001) or **C.** positive lymph node (*P* < 0.001) were significantly high in the non-responder group: **A.** *P* = 0.002; **B.** *P* < 0.001; **C.** *P* < 0.001.

After the data from the two studies was combined, three high-risk prognostic factors were explored, including vaginal surgical margin, parametrial infiltration and lymph node metastasis (Figure 8). All three high-risk prognostic factors occurred more frequently in the non-responder group, compared with the responder group (Supplementary Figure 2). The role of chemotherapy on responders’ post-surgery treatment was also shown in Supplementary Figure 3.

## DISCUSSION

This study combined data from a large case-control study and a prospective cohort study together to test the effect of the clinical response on long-term survival. In this study, clinical response was demonstrated to be an independent prognostic factor, as responders had significant higher DFS rates than non-responders.

It was discovered that being a clinical responder was a factor indicating a better DFS in the retrospective phase. This finding was also validated in patients with cervical cancer in a prospective cohort. Meanwhile, as NACT has been used in the treatment of cervical cancer for many years, previous studies have also revealed that short-term response is an indicator of long-term survival. Scholars (Xiong and colleagues) in Sun Yat-Sen University demonstrated that non-responders had relatively lower survival rates than responders [21]; others (Li and colleagues at Chongqing Medical University; Chen and colleagues at Wuhan University) also made the similar discovery [22, 23]. This study also validated other researchers’ findings as well as our previous results [19, 21–24]. Thus, we believe that early clinical response to chemotherapy can be treated as an indicator to DFS.

Our study also investigated a high-risk pathological factor, lymph node metastasis, as well as its relationship with DFS; our finding is consistent with previous findings. Li and colleagues discovered that patients without lymph node metastasis also achieved significantly longer survival [22], and Biewenga and colleagues demonstrated that lymph node metastasis led to worse overall survival [25]. Sevin and colleagues also revealed its prognostic effect on long-term survival [26]; Kamura and colleagues demonstrated that the presence of positive nodes had a negative effect on long-term survival with statistical significance [27]; and Lai and colleagues discovered that patients with positive nodes also achieved significantly shorter survival periods” [28]. Ho and colleagues also demonstrated that lymph node metastasis was an independent prognostic factor [29]; Alvarez and colleagues also revealed its prognostic effect on long-term survival [30]; Yuan and colleagues demonstrated that positive nodes led to worse overall survival [31]; and Deng and colleagues demonstrated that lymph node metastasis was a significant independent poor prognostic factor after multiple Cox regression analysis [32]. Thus, our research validated the previous findings of other scholars.

Our study also investigated high-risk factors, such as parametrial infiltration and vaginal surgical margin. Compared with responders, the high-risk factors were more frequently observed in the non-responders with statistically significance. We also validated the previous findings made by other scholars; Giaroli and colleagues also demonstrated that parametrial infiltration was an independent prognostic factor [33], as did other scholars [25, 34]. Furthermore, Hu and colleagues demonstrated that positive vaginal surgical margin had a negative effect on long-term survival with statistical significance [35], as did Suprasert and colleagues [36].

Although grade, cell type and LVSI were regarded as potential risk factors, our study failed to demonstrate these factors to be independent prognostic factors in multivariate Cox analysis. This is partly because NACT have eliminated the potential risk [19, 37], and thus, patients with G2/G3 differentiation, non-squamous cell type or positive LVSI also had excellent prognoses.

However, our study has some limitations. First, biomarkers that may contribute to the long-term survival of cervical cancer patients have not been investigated by us. Second, the reason why some patients achieved a clinical response but some patients did not has not been explored in this study. Third, a pathological response was also reported to be an important prognostic factor [38]; however, it was not evaluated in this study. In further studies, we will explore the role of biomarkers in survival, the reason why some women can achieve clinical response while others do not, and the role that the pathological response plays in long-term DFS.

In conclusion, our study revealed that the early response to NACT was a predictor of DFS using data from a retrospective study. This predictor was also validated in data from a prospective cohort study. This result may help doctors and patients to predict long-term survival. Further study should identify as many risk factors as possible and combine the risk factors together to give clinicians a more definite method for predicting long-term survival among patients with cervical cancer who are treated with NACT and surgery.

## MATERIALS AND METHODS

The information used in this study included data from a retrospective case-control study and a prospective cohort study (the registration number at Clinicaltrial.gov was NCT01628757). This study follows the Declaration of Helsinki and was carried out in accordance with the approved guidelines. All experimental protocols were approved by the ethical committee at Huazhong University of Science and Technology. All eligible patients gave written informed consent before entering this study.

### Eligibility criteria

The patients’ inclusion criteria were as follows: patients with cervical cancer diagnosed by pathologists and clinicians; age ≥ 18 years old and ≤ 70 years old; Karnofsky score > 70; normal cardiac function; normal EKG; normal chest X-ray; normal hepatic function with normal total bilirubin (≤ 1.5 × the upper normal limit); normal renal function with normal serum creatinine level (≤ 1.5 × the upper normal limit); WBC count ≥ 4 × 109/L and ≤ 10 × 109/L; absolute neutrophil count (ANC) ≥ 2 × 109/L; HB concentration ≥ 90 g/L; PLT count ≥ 100 × 109/L.

The patients were excluded for any of the following reasons: previously treated cervical cancer; history of other malignant tumor diseases; sepsis or other active infection; heart disease such as heart failure or myocardial infarction; withdrawing consent or leaving the study.

### Criteria to evaluate the clinical response

The criteria adopted in this study are the WHO standards, which were also adopted by the previous studies [19, 39, 40]. Responders were defined as CR (complete response) + PR (partial response). CR means the disappearance of all tumor cells; PR means a resident tumor size less than 50% of the primary tumor size, without new lesions; SD (stable disease) means the tumor size reduction is less than 50% of the primary size, without new lesions; PD (progressive disease) means chemotherapy is useless, and the tumor size increases by greater than 25% or new lesions appear even after chemotherapy.

Ultrasound of the tumor as well as the pelvic condition was scheduled and examined after each NACT cycle to control for progressive disease among all patients. If the tumors were considered to be operable, radical surgery was performed within 4 weeks after the completion of the last scheduled chemotherapy cycle. Otherwise, the patients underwent CCRT. After the completion of the safety follow-up (i.e., 4 weeks after surgery), the decision regarding systemic adjuvant post-surgery therapy was at the discretion of the treating gynecologists. Patients who had risk prognostic factors, such as parametrial infiltration, positive lymph nodes, deep stromal invasion, lymph vascular involvement, or positive surgical margin, received postoperative irradiation or postoperative chemotherapy.

### Patient follow-up

Patients were followed every three months in the first year, every six months in the second and third year, and every 12 months thereafter. The examination included pelvic MRI or computerized tomography (CT), pelvic ultrasound, physical examination of the pelvis by the gynecologist, chest X-ray, EKG, blood RT, renal function, and hepatic function.

### Statistical analysis

The Kaplan-Meier method was used to construct the survival curves for DFS. A log-rank test was used to compare the survival rate between the responders and the non-responders. A univariate Cox regression model was used to evaluate the role of all the variables, including clinical factors and pathological factors, as significant prognostic factors. A multivariate Cox regression model was used to identify the independent prognostic factors among both the clinical factors and the pathological factors. All statistical analysis was performed using the IBM SPSS 20.0 statistical software package.

## SUPPLEMENTARY MATERIALS FIGURES AND TABLES


