# Supplementary material for: Early response to neoadjuvant chemotherapy can help predict long-term survival in patients with cervical cancer
Source: Oncotarget. 2016 Aug 20;7(52):87485–95. doi: 10.18632/oncotarget.11460 (PMC5350004; doi:10.18632/oncotarget.11460)
Supplement: Supplementary file 1 [file oncotarget-07-87485-s001.pdf]

Early response to neoadjuvant chemotherapy can help predict long-term survival in patients with cervical cancer

Supplementary Material

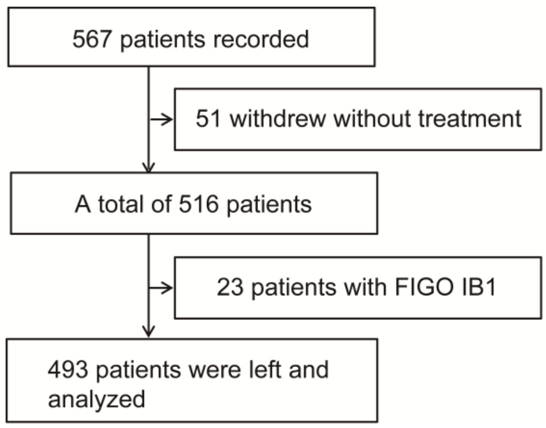

Supplementary Figure 1. Patients' flowchart in the prospective study.

Legend: A total of 516 patients were included in this study in the beginning; 23 patients with FIGO IB1 stage cervical cancer were excluded from further analysis; 493 patients with FIGO stage IB2-IIB were left and their data were finally analyzed.

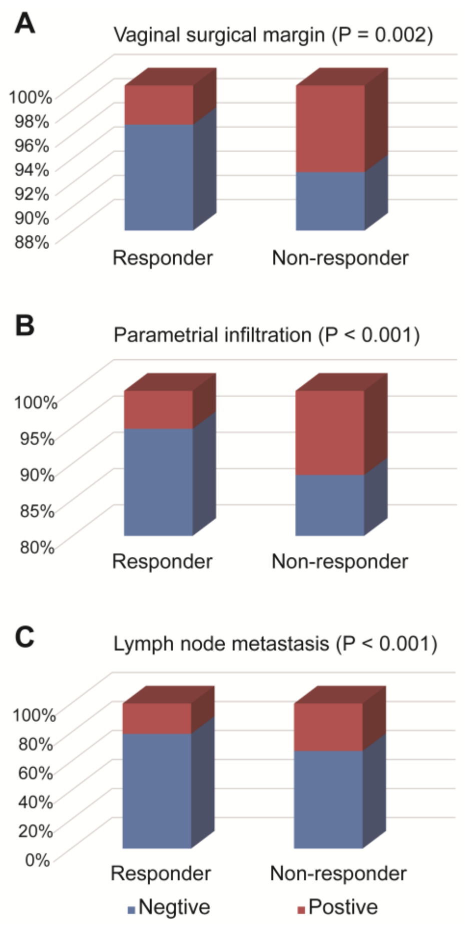

Supplementary Figure 2. High-risk prognostic factors' distribution among the responders and non-responders.

Legend: The ratio of cases with (A) positive vaginal surgical margin (P = 0.002), (B) positive parametrial infiltration (P < 0.001) or (C) positive lymph node (P < 0.001) were significantly high in the non-responder group: (A) P = 0.002; (B) P < 0.001; (C) P < 0.001.

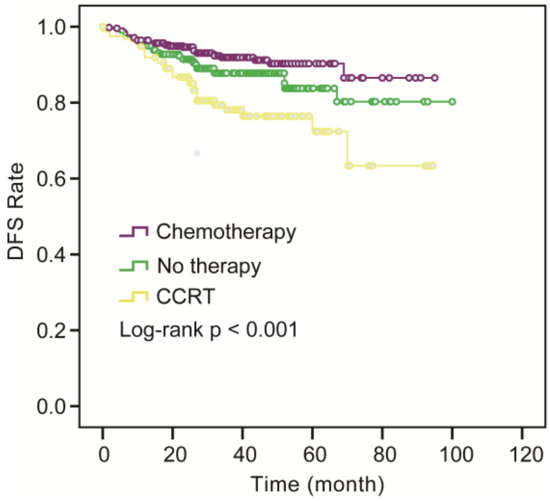

Supplementary Figure 3. Comparability of post-operative treatments among responders.

Legend: Chemotherapy led to significantly higher disease-free survival rate than any other therapy with $P < 0.001$. CCRT, concurrent chemoradiotherapy.

Supplementary Table 1. Clinical response rates in both studies according to WHO criteria.

Study	CR	PR	SD	PD	Unknown
**Retrospective**	61	576	183	6	27
**Prospective**	59	311	102	13	8

Supplementary Table 2. Univariate Cox regression for DFS in the retrospective study.

Variables		Retrospective		
		**HR**	**95%CI**	**P**
**Clinical response**	Non-responder vs Responder	1.83	1.18 to 2.85	0.007
**Age**	>44 vs ≤44 years	1.61	1.05 to 2.48	0.03
**Stage**	IIA vs IB2	2.18	1.13 to 4.20	0.02
	IIB2 vs IB2	2.44	1.33 to 4.48	0.004
**Tumor size**	>4cm vs ≤4cm	1.37	0.86 to 2.19	0.19
**Grade**	G2 vs G1	2.16	0.67 to 7.01	0.20
	G3 vs G1	3.38	1.06 to 10.81	0.04
	Undetermined vs G1	2.05	0.60 to 6.97	0.25
**Cell type**	Non-squamous vs squamous	2.24	1.32 to 3.82	0.003
**LVSI**	Positive vs negative	1.40	0.75 to 2.61	0.29
**Parametrial infiltration**	Positive vs negative	2.61	1.53 to 4.44	<0.001
**Vaginal surgical margin**	Positive vs negative	1.91	0.83 to 4.41	0.13
**Lymph node metastasis**	Positive vs negative	3.68	2.21 to 6.12	<0.001

LVSI, Lymph vascular space invasion. DFS, disease free survival.

Supplementary Table 3. Univariate Cox regression for DFS in the prospective study.

**Variables**		**Prospective**		
		**HR**	**95%CI**	**P**
**Clinical response**	Non-responder vs Responder	2.50	1.44 to 4.34	0.001
**Age**	>44 vs ≤44 years	2.21	1.24 to 3.96	0.008
**Stage**	IIA vs IB2	1.69	0.63 to 4.54	0.3
	IIB2 vs IB2	2.77	1.23 to 6.24	0.01
**Tumor size**	>4cm vs ≤4cm	1.02	0.56 to 1.89	0.94
**Grade**	G2 vs G1	0.57	0.15 to 2.15	0.41
	G3 vs G1	0.75	0.34 to 1.70	0.5
	Undetermined vs G1	1.11	0.49 to 2.55	0.8
**Cell type**	Non-squamous vs squamous	1.46	0.64 to 3.32	0.37
**LVSI**	Positive vs negative	2.79	1.00 to 7.82	0.05
**Parametrial infiltration**	Positive vs negative	3.15	1.13 to 8.81	0.03
**Vaginal surgical margin**	Positive vs negative	3.86	1.62 to 9.17	0.002
**Lymph node metastasis**	Positive vs negative	2.58	1.41 to 4.72	0.002

LVSI, Lymph vascular space invasion. DFS, disease free survival.

Supplementary Table 4. Multivariate Cox regression for DFS in the retrospective study.

**Variables**	**Retrospective**		
	**HR**	**95%CI**	**P**
**Clinical response**			
**Responder**	1		
**Non-responder**	1.59	1.01 to 2.50	0.046
**FIGO stage**			
**IB2**	1		
**IIA**	2.08	1.07 to 4.06	0.03
**IIB**	2.19	1.17 to 4.09	0.01
**Grade**			
**G1**	1		
**G2**	3.02	0.91 to 10.05	0.07
**G3**	5.00	1.50 to 16.62	0.009
**Undetermined**	2.78	0.80 to 9.63	0.11
**Cell type**			
**Squamous**	1		
**Non-squamous**	2.59	1.51 to 4.45	0.001
**Parametrial infiltration**			
**Negative**	1		
**Positive**	2.05	1.18 to 3.53	0.01
**Lymph node metastasis**			
**Negative**	1		
**Positive**	3.19	2.08 to 4.90	<0.001

FIGO, International Federation of Gynecology and Obstetrics.

Supplementary Table 5. Multivariate Cox regression for DFS in the prospective study.

**Variables**	**Prospective**		
	**HR**	**95% CI**	**P**
**Clinical response**			
**Responder**	1		
**Non-responder**	2.09	1.10 to 4.00	0.02
**Age (years)**			
**≤44**	1		
**>44**	2.35	1.26 to 4.37	0.007
**Vaginal surgical margin**			
**Negative**	1		
**Positive**	2.50	1.01 to 6.17	0.047
**Lymph node metastasis**			
**Negative**	1		
**Positive**	2.34	1.24 to 4.41	0.009

FIGO, International Federation of Gynecology and Obstetrics.
